# CUT&ID for simultaneous profiling of protein–DNA and protein–protein interactions

**DOI:** 10.1093/nar/gkag943

**Published:** 2026-09-30

**Authors:** Anna Nordin, Claudio Cantù

**Affiliations:** Wallenberg Centre for Molecular Medicine, Linköping University, SE-581 83 Linköping, Sweden; Department of Biomedical and Clinical Sciences, Division of Molecular Medicine and Virology, Faculty of Medicine and Health Sciences, Linköping University, SE-581 83 Linköping, Sweden; Science for Life Laboratory, Linköping University, SE-581 83 Linköping, Sweden; Wallenberg Centre for Molecular Medicine, Linköping University, SE-581 83 Linköping, Sweden; Department of Biomedical and Clinical Sciences, Division of Molecular Medicine and Virology, Faculty of Medicine and Health Sciences, Linköping University, SE-581 83 Linköping, Sweden; Science for Life Laboratory, Linköping University, SE-581 83 Linköping, Sweden

## Abstract

We developed CUT&ID to simultaneously profile the native genomics and proteomics associations of any endogenous target, in a single workflow with no need for transgenesis. CUT&ID is enabled by a fusion protein of TurboID, protein A/G, and micrococcal nuclease, which drives sequential target recognition, proximal protein biotinylation, and DNA cleavage in living cells. CUT&ID is broadly applicable, works on fresh, frozen, or fixed nuclei, and provides high signal-to-noise genomics and proteomics identification across all types of gene regulators tested, including histone modifications, transcription factors, and non-DNA-binding proteins. CUT&ID’s streamlined protocol yields a dual readout from 1 million cells per sample, making it an attractive alternative to existing methodologies.

## Graphical Abstract

**Graphical Abstract:**
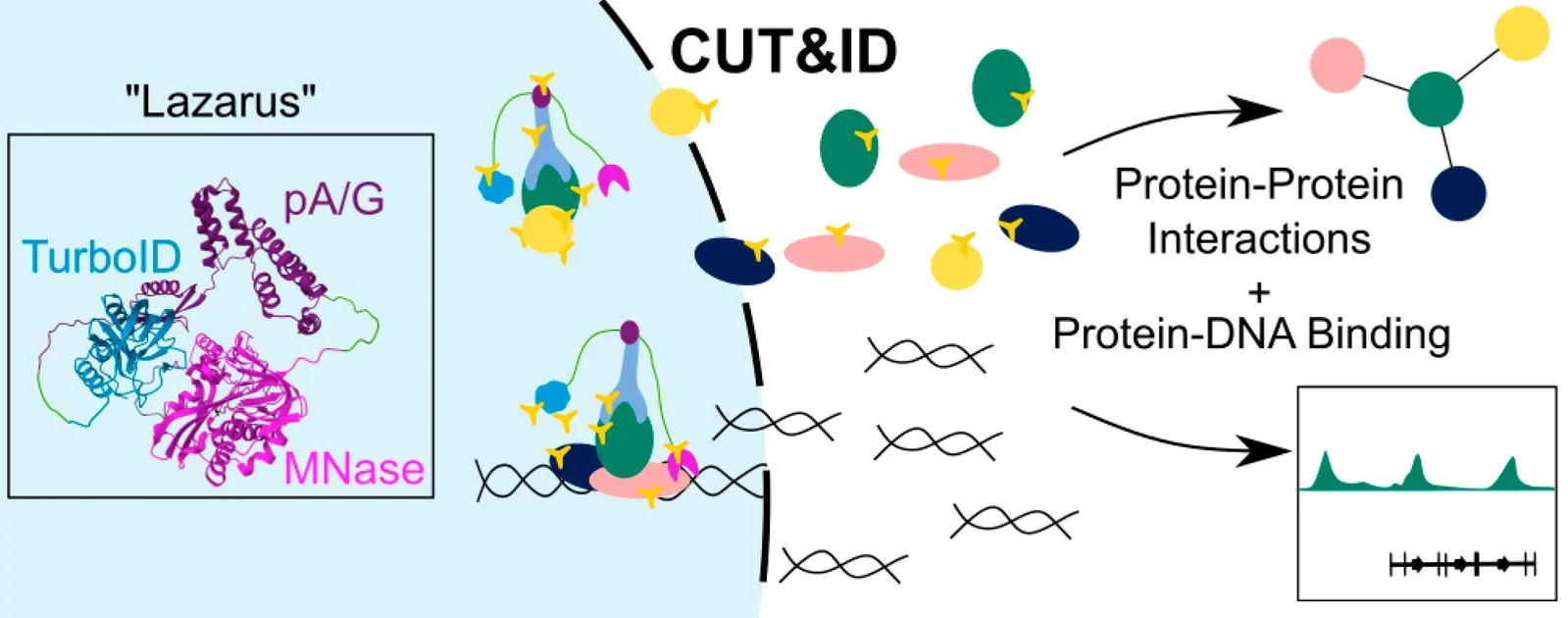

## Introduction

Gene expression drives cell fate specification and cell identity, and its dysregulation is a hallmark of essentially every disease. This process involves a complex system of transcription factors (TFs) and co-factors that, in addition to associating to genomic regulatory regions, work together to activate or inhibit transcription [1]. Both of these aspects are crucial to understanding gene regulation. Recent method advancements have enabled increasingly detailed investigation of these two dimensions, offering improved sensitivity and reduced input, though these two scopes have remained largely separated [2–9].

Methods to profile protein–DNA interactions have evolved substantially from early chromatin immunoprecipitation (ChIP) approaches. ChIP-seq [2] remains a staple technique, enabling genome-wide mapping of protein binding sites, but typically requires large cell numbers and relies on formaldehyde crosslinking, which can introduce artifacts and background noise. More recent methods such as CUT&RUN [3] and CUT&Tag [4] improve resolution and reduce input requirements by using targeted enzymatic cleavage or tagmentation at binding sites, minimizing background and enabling profiling in low cell numbers. CUT&Tag is applicable even to single cells, though buffer conditions make it less suitable for most TFs and non-DNA-binding proteins. We developed CUT&RUN LoV-U [5], further optimized for transcriptional co-factors, which are often difficult to capture. Despite these advantages, these techniques are limited to mapping genomic occupancy and do not directly provide information about protein interaction partners.

Complementary advances in proteomics have enabled the identification of protein–protein interactions within chromatin-associated complexes. Approaches such as RIME [6] combine immunoprecipitation with mass spectrometry to identify interaction partners of a target protein, but like ChIP-seq, depend on crosslinking and substantial input material. Proximity labeling methods, including TurboID [7], allow labeling of proteins in the vicinity of a bait protein in living cells, enabling detection of transient or weak interactions. However, TurboID requires genetic tagging. The fusion of Protein A with TurboID (ProtA-Turbo [8]) overcame this limitation and allowed to target endogenous proteins for the first time, though it still requires a large input of cells, and to link protein interactions to specific genomic loci, ChIP-seq must be performed in parallel. Recently, PLAM-seq [9] was developed to obtain a combined proteomics and genomics readout, though it too requires tagging of the bait protein and cell fixation.

Here, we develop CUT&ID, a method that allows the simultaneous profiling of both the DNA binding and protein partners of all classes of gene regulators (histone marks, TFs, non-DNA-binding proteins), without crosslinking, tagging, and with 1 million cells per sample. The CUT&ID workflow is performed in 3 days, allows multi-channel pipette scale-up for increased throughput, and is successful across the board of all gene regulators tested.

## Materials and methods

### AlphaFold modeling

Structural predictions were done with ColabFold (v1.5.3) [10], with default settings and visualized and annotated using the Mol* Viewer software [11].

### Lazarus protein production

The codon-optimized sequence for TurboID-pAG-MNase (Lazarus) was synthesized by GenScript and cloned into the pET-28a(+)-TEV vector. The plasmid is being deposited in Addgene, and the Cantù lab is willing to share test aliquots of the plasmid or purified protein upon request.

One nanogram of plasmid was transformed into BL21 (DE3) (Cat. #70235, Sigma Aldrich) *Escherichia coli* cells and plated on kanamycin agar plates overnight. Colonies were picked and grown first for 16 h in 10 ml of LB broth, then 1 ml was taken and added to 200 ml of LB and grown until optical density (OD) reached 0.4. IPTG (Isopropyl β-D-1-thiogalactopyranoside) was added to a final concentration of 100 µM and the protein was produced for 20 h at RT (20°C) shaking. Bacteria were pelleted at 3000 × *g* at 4°C for 15 min and then washed twice in 20 ml ice-cold phosphate buffered saline (PBS). Bacteria were resuspended in 20 ml of 2× lysis buffer (300 mM NaCl, 20 mM Tris–HCl, pH 7.4, 40 mM imidazole, 0.1 mM DTT (dithiothreitol), 0.005% IGEPAL, 0.1 mM PMSF (phenylmethylsulfonyl fluoride)) and resuspended by vortexing. Lysis was completed by sonication for 5 min in a tip-sonicator, 5 s ON/OFF, amplitude 10. Lysate was cleared by centrifugation at max speed for 15 min at 4°C. Two-hundred fifty microliters of HisPur Ni-NTA resin (Cat. #88221, Thermo Scientific) was washed in 1× lysis buffer twice and then added to the lysate and incubated for 30 min in ice. The resin was pelleted by centrifugation at 500 × *g* for 2 min and then washed twice in 20 ml of 1× lysis buffer and then resuspended once in 500 µl of 1× lysis buffer. Ten microliters TEV protease (Cat. #P8118S, NEB) was added and incubation proceeded overnight at 4°C on a rotator. After incubation, the supernatant was kept and mixed with 500 µl of 100% glycerol for storage. The resin was resuspended in 500 µl of 1× lysis buffer with 250 mM imidazole for elution and mixed with 500 µl of 100% glycerol for storage. Cleaved and eluted fractions were quantified and visualized with sodium dodecyl sulfate–polyacrylamide gel electrophoresis (SDS–PAGE) followed by PageBlue staining, and the cleaved fraction was used for experiments. In standard conditions of SDS–PAGE, Lazarus may run as two bands of the expected size of ∼80 kDa, likely reflecting incomplete protein reduction.

### Enzymatic testing

DNA cleavage was assessed by combining plasmid DNA with either EDTA (ethylenediaminetetraacetic acid) or CaCl_2_ and Lazarus protein in PBS, followed by incubation for 30 min at 37°C for cleavage, and 5 min at 70°C for inactivation. Results were visualized on a 1% agarose gel with SYBR safe stain and visualized on the ChemiDoc (BioRad). Protein biotinylation was tested by lysing HCT116 nuclei in RIPA buffer with sonication (input) and then comparing this with release fraction from a targeted sample (target was SMARCA4; see the “CUT&ID” section below) versus the IgG control via streptavidin-HRP western blotting. Samples were combined with 2× Laemmli, boiled at 95°C for 10 min, and run on a 10% polyacrylamide gel during SDS–PAGE at 120 V for 90 min. Proteins were transferred to a nitrocellulose membrane with wet transfer overnight at 4°C at 30 V. Total protein content was visualized with Ponceau staining. The membrane was blocked for 1 h at room temperature (RT) with 5% bovine serum albumin TBS 0.1% Tween (TBST). The membrane was washed thrice with TBST and then incubated with Streptavidin-HRP (Cat. #21 130, Thermo Scientific) diluted 1:5000 for 1 h at RT and then washed six times. Pierce ECL western blotting substrate (Cat. #32 106, Thermo Scientific) was used for detection and visualized on the ChemiDoc (Bio-Rad).

### Cell culture

HCT116 human male colorectal cancer cells were cultured in a 37°C incubator in 5% CO_2_ with humidity. Culture medium consisted of high glucose Dulbecco’s modified Eagle’s medium (Cat. #41965039, Gibco) with 10% fetal bovine serum (Cat. #26140079, Gibco) and 1× PenStrep (Cat. #15140148, Gibco). One million cells/sample were harvested by incubation with Trypsin-EDTA (Cat. # 25200056, Gibco) for 5 min. The trypsin was quenched with culture media, and the cells were washed twice with DPBS (Cat. #14190094, Thermo Fisher Scientific). For fixed samples, cells were resuspended in 0.1% formaldehyde in PBS and incubated at RT for 10 min. Fixation was quenched with 250 mM glycine.

### CUT&ID

Nuclear extraction was performed by three washes with nuclear extraction (NE) buffer (20 mM HEPES–KOH, pH-8.2, 10 mM KCl, 0.5 mM spermidine, 0.05% IGEPAL, 20% glycerol, 0.1 mM PMSF). For frozen nuclei, after the third wash, all supernatant was removed and pellets were frozen in an isopropyl chamber and stored at −80°C. The nuclei were resuspended after the final wash in 1 ml wash buffer (20 mM HEPES, pH 7.5, 150 mM NaCl, 0.5 mM Spermidine, 0.1 mM PMSF) per sample and bound to 40 µl Magnetic ConA beads (Cat. #93569, Cell Signaling Technology) equilibrated in provided binding buffer. Bead binding proceeded for 15 min at 4°C, after which nuclei and beads were resuspended in 200 µl wash buffer per sample and split into PCR (polymerase chain reaction) tubes. Beads were collected on the magnet and then resuspended in 200 µl EDTA wash buffer (wash buffer with 2 mM EDTA) and incubated at RT for 5 min. Samples were then directly resuspended in antibody buffer (wash buffer with 2 µg antibody, 2 mM EDTA, 0.025% digitonin, and 0.05% BSA) and incubated overnight at 4°C on a rotator. Antibodies used included anti-CTCF (abcam, ab188408), anti-SMARCA4 (antibodies-online, ABIN6991990), anti-H3K27ac (antibodies online, ABIN2668475), anti-TCF7L2 (Cell Signaling Technology, C48H11), anti-β-catenin (antibodies online, ABIN2855042), anti-JUN (antibodies-online, ABIN3020286), anti-RAD21 (antibodies-online, ABIN2856242), anti-HDAC1 (antibodies-online, ABIN2854776), and rabbit IgG isotype control (Invitrogen, #100500C).

After overnight incubation samples were washed five times (wash buffer with 0.025% digitonin) and resuspended in 200 µl of Lazarus protein buffer (wash buffer with 480 ng Lazarus/sample) and incubated for 1 h at 4°C on a rotator. Next, samples were washed five times and then resuspended in 100 µl biotinylation buffer (wash buffer with 5 mM MgCl_2_, 5 µM biotin, and 2 mM ATP) and incubated at 37°C for 30 min. Samples were washed twice to remove excess biotin, chilled in ice for 5 min during the last wash, and then resuspended in 100 µl ice-cold digestion buffer (wash buffer with 2 mM CaCl_2_). Digestion was allowed to proceed for 30 min at 4°C. Digestion was stopped by the addition of STOP mix (1 µl of 500 mM EDTA, 1 µl of 500 mM EGTA, and 4 µl of 5M NaCl). Samples were incubated at 37°C for 30 min and then microcentrifuged for 30 s. The first release was transferred to new PCR tubes, and the beads resuspended in 50 µl of 1× Urea STOP buffer (100 mM NaCl, 2 mM EDTA, 2 mM EGTA, 0.5% IGEPAL, 8.6 M Urea) and incubated for 30 min at 20°C. After incubation, samples were microcentrifuged for 30 s and then the supernatant was combined with the first release and the beads were discarded. 20 µl Pierce streptavidin beads (Cat. #88817, Thermo Scientific) were prepared per sample by washing three times in PBS and then resuspended in 50 µl PBS with 0.1 mM PMSF. Beads were added to each sample (final volume 200 μl) and incubated at RT for 1 h on a rotator. The beads were collected on a magnet, and supernatant containing DNA and non-biotinylated proteins were processed for DNA extraction (see the “DNA sequencing” section) and the beads attached to biotinylated proteins were processed for mass spectrometry (see the “mass spectrometry” section).

### DNA sequencing

Supernatant from Pierce beads was transferred to new tubes containing 2 µl of 10% SDS and 2.5 µl proteinase K, and incubated at 50°C for 1 h. One volume of phenol:chloroform:isoamyl alcohol was added to each sample and vortexed for 30 s. Samples were centrifuged at 15 000 × *g* for 15 min, and then the aqueous phase was transferred to new tubes containing 1 µl glycogen, 20 µl of 3M sodium acetate, and 500 µl of 100% EtOH. Samples were incubated overnight at −20°C, centrifuged at 15 000 × *g* at 4°C for 45 min, and the supernatant was discarded. Pellets were washed once with 1 ml of 70% EtOH, centrifuged again for 10 min, and then dried for 10 min at 50°C. Pellets were resuspended in 15 µl of 10 mM Tris–HCl, pH 8.0. Library preparation was performed with the KAPA EvoPrep kit (Cat. #10096039001, Roche) and KAPA dual-index adapters according to manufacturer’s instructions with the following modifications: 0.5× volumes were used throughout the protocol, libraries were amplified for 13 PCR cycles, and samples were size-selected by cutting from 150–500 bp after running on 2% agarose E-gel EX gels (Cat. #G8142ST, Invitrogen) with the Thermo Fisher E-Gel System. Samples were sequenced with 50 bp pair-end reads on the Illumina NextSeq 2000 with the NextSeq 1000/2000 P2 XLEAP RGTSKit (100c) to ~5–10 million raw reads/sample.

### Genomic data analysis

Reads were trimmed with bbmap bbduk (version 38.18) [12] to remove adapters and poly [AT], [G], and [C] repeat sequences. Reads were aligned to the hg38 genome with bowtie2 (version 2.4.5) [13] with the options --local --very-sensitive-local --no-unal --no-mixed --no-discordant --phred33 --dovetail -I 0 -X 500. Samtools (version 1.11) [14] suite was used to remove duplicate and incorrectly paired reads. Bedtools (version 2.30.0) [15] was used to remove reads mapped to the CUT&RUN hg38 suspect list from bam files. Bigwigs were created for visualization using deepTools (version 3.5.1-0) [16] with the options -e and -RPCG. After quality control (QC) of individual datasets, biological duplicates were merged using samtools. Peaks were called on merged datasets using MACS2 [17] with the options --keep-dup all, -f bampe, and -q 0.05. Heatmaps, signal intensity plots, and QC plots were created with deepTools. Motif analysis was done using HOMER (version 4.11) [18] findMotifsGenome with the hg38 genome using -size 200. Peaks for ENCODE CTCF ChIP-seq in HCT116 were downloaded for comparison (ENCSR240PRQ) [19]. Genome browser images were created with IGV [20] with RPCG normalized bigwigs.

### Mass spectrometry

Pierce streptavidin beads were washed five times in protein wash (PW) buffer (10 mM Tris–HCl, pH 7.5, 150 mM NaCl, 0.1% sodium deoxycholate, 0.1% SDS, 1% Triton X, 1 mM EDTA, 0.5 mM EGTA), once in 1 M KCl, once in 0.1 M Na_2_CO_3_, twice in PW buffer, transferred to a new tube, once in 2 M urea in 50 mM Tris–HCl, pH 7.5, once in 50 mM Tris–HCl, pH 7.5, transferred to a new tube, and then thrice in 50 mM ammonium bicarbonate (ABC). All wash volumes were 200 μl. Beads were resuspended a final time in 100 µl ABC and transferred to new low bind tubes, and then 0.4 µg of Pierce Trypsin protease (Cat. #90057, Thermo Scientific) was added per sample and incubated for 1 h at 37°C. The supernatant was transferred to a new tube, and the beads were washed twice with 50 µl ABC and the supernatants were pooled (final volume 200 μl). Beads were discarded. Samples were centrifuged at 15 000 × *g* for 10 min, and then 180 µl was transferred to new tubes containing 10 µl of 100 mM DTT and incubated for 30 min at 37°C. 26 µl of 100 mM iodoacetamide were added and samples were incubated at RT for 45 min in the dark. An additional 0.5 µg of trypsin was added and the samples were incubated overnight at 37°C on a rotator. After overnight incubation, samples were dried in a vacuum centrifuge, reconstituted in 40 µl resuspension solution (0.5% TFA, 5% ACN) and desalted using Pierce C18 columns (Cat. #89870, Thermo Scientific) according to manufacturer’s instructions. After desalting, samples were dried in a vacuum centrifuge and reconstituted in 0.1% formic acid. LC-MS/MS analysis was performed using a High Resolution Trapped Ion Mobility timsTOF HT mass spectrometer coupled to a nanoElute 2 LC system via a CaptiveSpray ionization source (Bruker Daltonics). Two hundred nanograms of peptide mixture sample reconstituted in 0.1% FA was loaded on reverse-phase C18 HPLC column (PepSep XTREME with dimensions of 25 cm x 150 µm x 1.5 µm, Bruker Daltonics) and peptides were separated in 45 min with a gradient of 0.1% formic acid in water (A) and 0.1% formic acid in acetonitrile (B) as follows: from 2% B to 17% B over 25 min; from 25% B to 37% B over 35 min; and from 37% B to 95% B over 45 min with a flow rate of 400 nl/min and a column temperature of 50°C. All data were acquired under the dia-PASEF mode with an MS1 scan range of 100–1700 *m*/*z*, and the collision energy was linearly interpolated between 1/K0 values, from 20 eV at 0.6 Vs/cm^2^ to 59 eV at 1.6 Vs/cm^2^, keeping constant above or below.

### Proteomics data analysis

dia-PASEF data files were analyzed in Spectronaut (version 20.2, Biognosys) using the human proteome FASTA with the addition of streptavidin, Lazarus enzyme, and rabbit IgG heavy chain. The following parameters were applied for the search: trypsin as the proteolytic enzyme, allowing up to one missed cleavage; carbamidomethylation of cysteine as a fixed modification; oxidation of methionine as a variable modification, and minimum peptide length of 5. FDR was set to 0.05 and normalization applied with default settings. Protein quantities, run evidence count, and number of precursors were exported as a protein report, and then filtered for keratins, tubulins, and ribosomal proteins. For differential analysis, protein quantities were uploaded to amica [21] and processed with default settings. Amica was used to visualize results and create figure panels. Samples for Figs 2 and 4 were processed with Spectronaut and analyzed with amica all together, while samples for Fig. 3 (fresh, frozen, fixed comparison) were processed separately and compared with amica’s compare datasets function. The mass spectrometry proteomics data, both raw files and .sne output have been deposited to the ProteomeXchange Consortium via the PRIDE partner repository with the dataset identifier PXD071610.

## Results

We designed a fusion protein containing protein A/G (pAG) for antibody recognition, micrococcal nuclease (MNase) for DNA under target cleavage, and TurboID for proximal protein biotinylation. We first used AlphaFold [22] to model different possible configurations and chose Nter-TurboID-pAG-MNase-Cter (hereafter referred to as Lazarus), carrying two intervening flexible polypeptide linkers to ensure proper folding and functionality of all domains (Fig. 1A). Lazarus can be expressed in bacteria and purified via standard protocols, yielding a protein of ~80 kDa (Fig. 1B). During protein production, endogenous biotin in the bacteria usually leads to Lazarus biotinylating itself (Fig. 1B), the extent of which may vary from batch to batch but does not affect downstream usage. Lazarus displays robust enzymatic activities *in vitro*: MNase cleaves DNA upon addition of CaCl_2_ (Fig. 1C, left). More Lazarus is retained in the nucleus when tethering with a targeted antibody for an abundant protein versus an isotype IgG (Fig. 1C, right), indicating the pAG is able to bind antibodies, and TurboID biotinylates itself and other surrounding proteins upon addition of biotin and ATP (Fig. 1C, right). We optimized the CUT&ID workflow via gradual tinkering starting from combined CUT&RUN LoV-U [5] and TurboID [7] protocols (Fig. 1D; detailed step-by-step protocol with troubleshooting help in Supplementary File S1).

**Figure 1.**
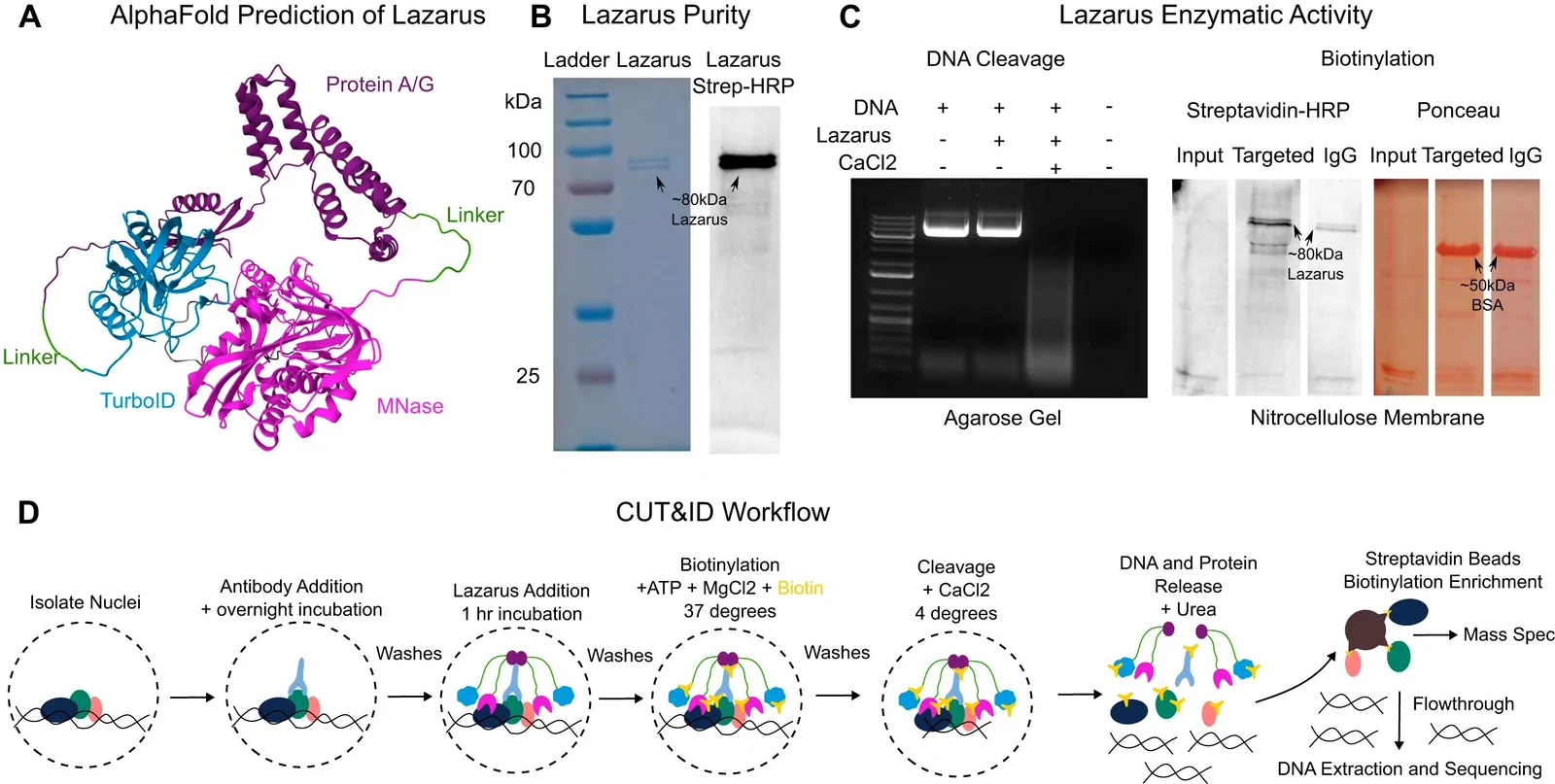
Lazarus activity and CUT&ID workflow. (**A**) AlphaFold predicted structure of Nter-TurboID-pAG-MNase-Cter recombinant protein (referred to as Lazarus), containing from N to C termini: TurboID (blue), linker (green), protein A/G (purple), linker (green), and MNase (pink). (**B**). Purity of Lazarus as seen by PageBlue staining of an SDS–PAGE gel (left; Lazarus typically runs as two close bands at ∼80 kDa, potentially due to incomplete denaturation/reduction or post-translational modification). Lazarus biotinylates itself during protein production, which can be seen by western blotting with streptavidin-HRP (right). (**C**) Enzymatic *in vitro* assays showing that Lazarus cleaves DNA upon CaCl_2_ addition (left, DNA agarose gel), that it is retained by target antibodies during the CUT&ID workflow and biotinylates itself and other proteins after addition of biotin and ATP (right, streptavidin-HRP western blot, compare target to IgG control). Ponceau stained membrane on the right shows total protein. Note that the input (nuclear lysate) does not contain BSA. (**D**) Schematic depiction of the CUT&ID workflow.

We first benchmarked CUT&ID on the two broadly relevant genome regulators: CTCF and SMARCA4/BRG1, comparing against isotype IgG negative control antibody added at the same amount (2 µg/sample). The genomics data of individual replicates (*n* = 2) were merged after QC, and peaks called with MACS2 against the IgG. For proteomics, we performed two technical replicates for each biological replicate. The proteomic data were processed with Spectronaut for identification, quantification, and normalization, and then with amica [21] on default settings for imputation and differential enrichment analysis. Peaks are listed in Supplementary Table S1, and enriched proteins in Supplementary Table S2. Protein quantity and precursor information from Spectronaut is provided in Supplementary Table S3, and amica output data for all datasets in Supplementary Table S4, which can be loaded into the online tool for data exploration. QC metrics of all genomic datasets including PCA, Spearman’s correlation, FrIP scores, and peak overlaps are provided in Supplementary Fig. S1A–D, showing high concordance between replicates, which cluster together on PCA and separate from IgG, and similar FrIP scores between replicates. QC metrics of all proteomics, including identified proteins, missing values, imputed intensities, coefficient of variation, PCA, correlation, and identification overlaps, are detailed in Supplementary Fig. S2A–G (for results presented in Fig. 2 and 4) and Supplementary Fig. S3A–G (for results presented in Fig. 3). Technical replicates (*n* = 2) show more consistency than the biological replicates (*n* = 2), but targets separate well based on PCA and Pearson’s correlation on imputed intensity. Experimental runs/batches varied in number of proteins identified, best reflected by the baseline proteins identified in the IgG negative controls.

**Figure 2.**
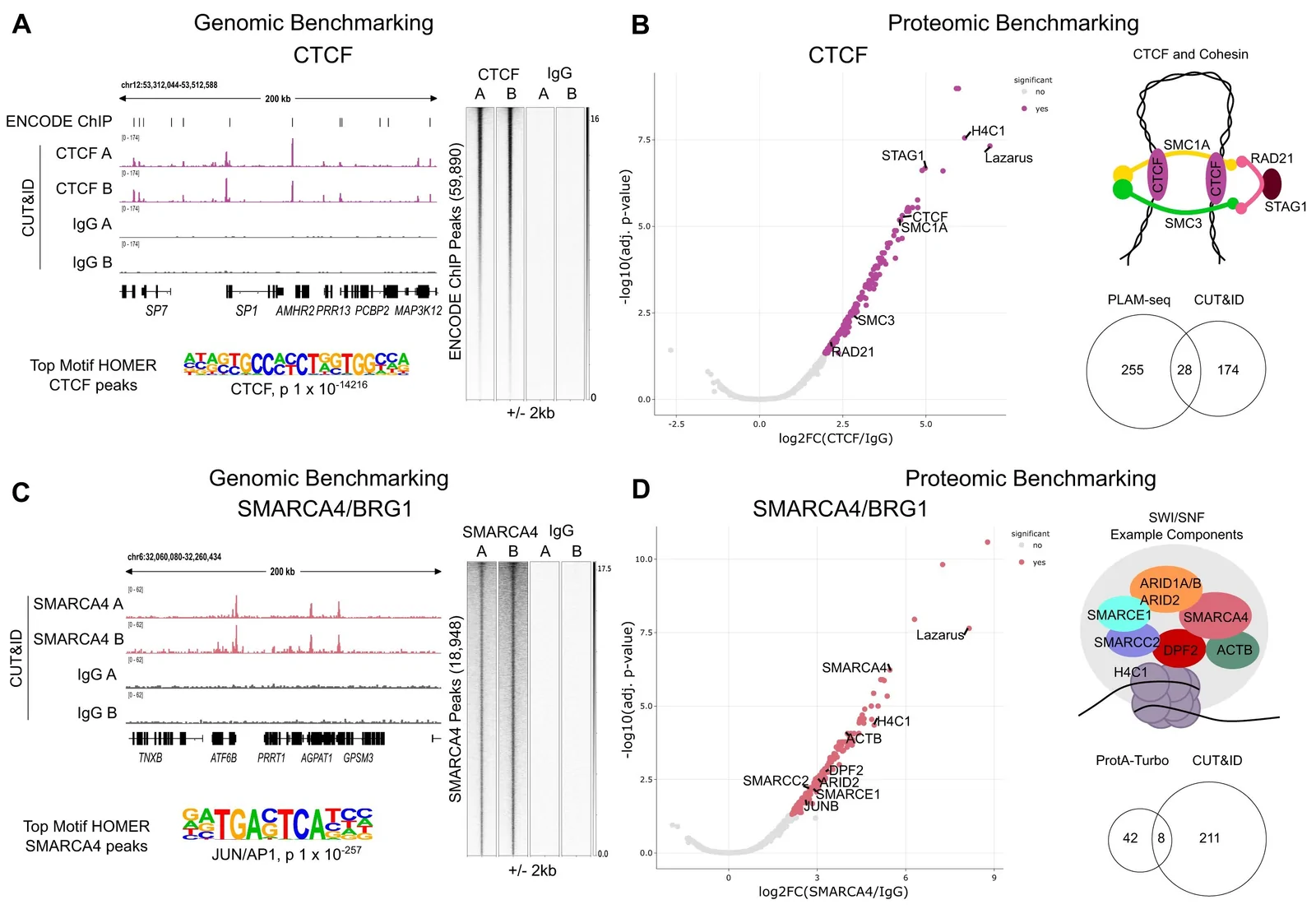
Benchmarking CUT&ID on CTCF and SMARCA4. (**A**) Genomic benchmarking of CTCF, showing IGV browser tracks of CUT&ID for CTCF, IgG, and ENCODE ChIP-seq peaks (top), CTCF as the top enriched HOMER motif (bottom), and high signal within all ENCODE ChIP-seq peaks (right). (**B**) Proteomic benchmarking of CTCF represented as volcano plot of CTCF/IgG enrichment (left) and the recovery of “textbook” interactors (cohesin complex schematic, top right). Venn diagram of enriched protein overlap with PLAM-seq on CTCF in HeLa cells is shown in the bottom right. (**C**) Genomic benchmarking of SMARCA4, showing genome browser view (top), JUN/AP1 as the top enriched motif (bottom), and signal enrichment within called peaks (right). (**D**) Proteomic benchmarking of SMARCA4, showing volcano plot of SMARCA4/IgG enrichment (left) and the recovery of “textbook” interactors, including SWI/SNF complex members (schematic in top right). Venn diagram of enriched protein overlap with ProtA-Turbo done on SMARCA4 in HeLa cells is shown in the bottom right. Peaks were called on merged replicates with MACS2 *q* < 0.05. Genomic datasets were normalized by reads per genome coverage and group scaled. Proteomic significance calculated using amica on default settings, with Log_2_FC > 1, adj. *P* < .05. PLAM-seq and ProtA-Turbo enriched protein lists were downloaded and filtered with the same significance thresholds used for CUT&ID.

**Figure 3.**
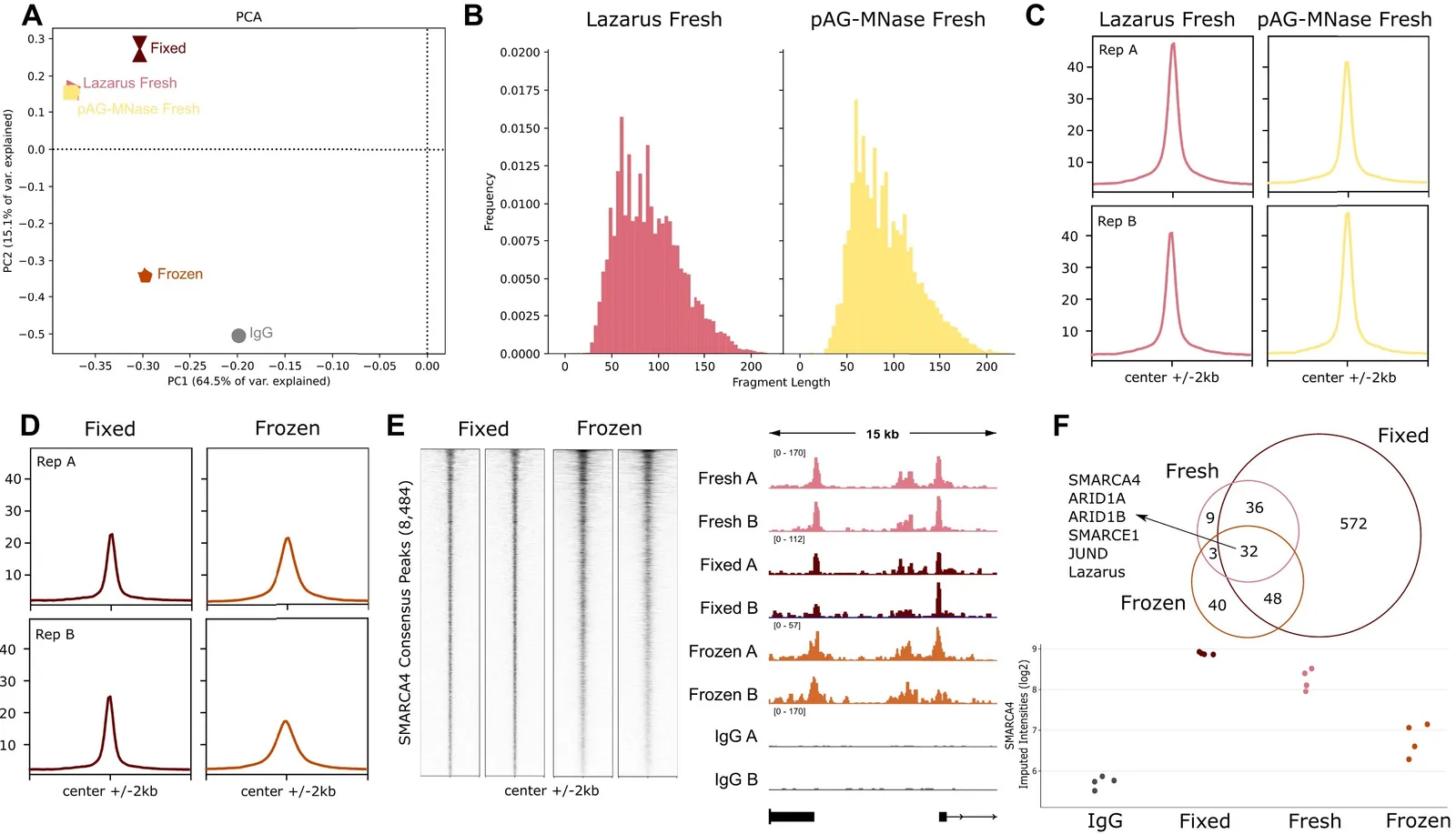
CUT&ID comparison with CUT&RUN. (**A**) PCA plot of genomic datasets. Samples from the Lazarus and pAG-MNase enzymes clustered together. (**B**) Comparison of fragment lengths of SMARCA4 samples using Lazarus versus pAG-MNase. Distributions were similar. (**C**) Comparison of average signal profiles of both replicates targeting SMARCA4 with Lazarus versus pAG-MNase. Average profiles are shown over the consensus peaks, defined as peaks called in at least three of the four datasets. (**D**) Signal profiles of Lazarus targeting SMARCA4 in fixed material (0.1% formaldehyde for 10 min) versus frozen (nuclear pellet frozen and then thawed for CUT&ID). Signal profiles are generally lower than those from fresh samples [panel (C)]. (**E**) Signal heatmaps (left) and IGV browser views (right) of fresh, fixed, and frozen samples. While signals are lower, fixed, and frozen samples still show enrichment within the consensus peaks. (**F**) Top: Venn diagram of the overlap of enriched proteins between the fresh, fixed, and frozen samples. All were enriched for SMARCA4 and various SWI/SNF complex members. Bottom: imputed intensities (Log_2_) of SMARCA4 compared to the IgG. Peaks were called with MACS2 *q* < 0.05. Genomic datasets were normalized by reads per genome coverage and scaled by group. Proteomic significance calculated using amica on default settings, with Log_2_FC > 1, adj. *P* < .05.

The CTCF genomics datasets exhibit the hallmarks of a successful CUT&RUN: high signal to noise ratio (FrIP scores 12% and 14%, 34 941 called peaks at MACS2 *q* < 0.05), enrichment of the primary motif (CTCF, *P* 1 × 10^−14 216^), and a high degree of overlap with ENCODE ChIP-seq data [19] (71% of CUT&ID peaks was shared with ChIP-seq, and strong signal from CUT&ID was exhibited across ENCODE peaks) (Fig. 2A). In the proteomics part we identified 202 enriched proteins over the IgG control (Log_2_FC > 1, adj. *P* < .05), including CTCF itself (Log_2_FC 3.9, adj. *P* 3.0 × 10^−5^) and known interactors like STAG1, SMC1A, SMC3, and RAD21 (Fig. 2B, left, schematic depiction of CTCF and the enriched cohesin subunits on the upper right) [23]. Many fold changes were calculated based on imputed values because in the IgG samples some of these proteins were not detected at all in this batch. Next, we compared the recently developed method PLAM-seq with CUT&ID. Filtered by us with the same thresholds, PLAM-seq for CTCF yielded 283 enriched proteins, 28 of which overlapped with CUT&ID (Fig. 2B, lower right), including all of the cohesin subunits listed above [9]. PLAM-seq was performed in HeLa, while CUT&ID was done in HCT116 cells. Notably, CUT&ID identified important known interactors of CTCF missed by PLAM-seq, including RNA Pol II, PARP1 [24], and DDX5 [25].

The SMARCA4 experiment was similarly successful, with high signal-to-noise ratios for both genomics and proteomics identifications (Fig. 2C and D). We identified 18 948 genomic binding sites (MACS2 *q* < 0.05), with JUN/AP1 as the highest enriched consensus motif (Fig. 2C), and 219 enriched proteins, including SMARCA4 (Log_2_FC 5.2, adj. *P* 1.3 × 10^−6^), JUN, and other members of the SWI/SNF complex (ARID2, SMARCC2, SMARCE1, and DPF2) (Fig. 2D, left, schematic depiction in upper right) [26]. We compared CUT&ID with ProtA-Turbo [8] for SMARCA4. Filtered with the same significance thresholds, ProtA-TurboID from 15 million unfixed cells resulted in 50 enriched proteins, 8 of which overlapped with CUT&ID (Fig. 2D, lower right), mostly SWI/SNF complex members. Both techniques had non-overlapping SWI/SNF identifications, potentially implicating diversity in the prevalence of different BAF complexes across cellular contexts. ProtA-Turbo was performed in HeLa cells (CUT&ID in HCT116). Other technical factors, such as the use of a different antibody targeting SMARCA4, may also underlie some of the differences.

To directly compare CUT&ID and CUT&RUN, we repeated the SMARCA4 experiments, this time either using Lazarus or pAG-MNase, with identical experimental conditions and from the same number of cells. We also wanted to test whether CUT&ID would be applicable to material that cannot be prepared fresh, so we included nuclei that were frozen after nuclear extraction, and nuclei from cells that were fixed with formaldehyde prior to extraction (0.1% formaldehyde for 10 min). On a PCA, the Lazarus samples clustered together with the pAG-MNase samples, while the other conditions and the IgG were clearly separated (Fig. 3A). When we compared the fragment size distribution of Lazarus samples versus the pAG-MNase samples, there were no apparent differences based on the enzyme used (Fig. 3B). Both enzymes resulted in comparable number of peaks (Lazarus had 14 092 and 10 354; pAG-MNase had 10 383 and 10 305), and the overlap between replicates was similar to overlap between enzymes, with 8484 peaks called in at least three of the four datasets (consensus peaks). FrIP scores on the consensus peaks were consistent (3.7% and 3.2% for Lazarus; 3.2% and 3.7% for pAG-MNase), and average signal profiles indistinguishable (Fig. 3C). Taken together, this data proves that on a genomic level, Lazarus performs comparably to pAG-MNase and is not hindered by its greater size due to the addition of the TurboID domain.

Both the fixed and frozen samples showed lower efficiency compared to the fresh samples: this was reflected in lower FrIP scores within the previously defined consensus peaks (1.8% and 1.6% for fixed; 2.2% and 1.8% for frozen) and signal to noise ratios (Fig. 3D). However, they recovered >50% of the consensus peaks from the fresh samples, and inspection of signal heatmaps and genomic loci indicate that, while less efficient than fresh samples, the genomic portion of CUT&ID can be satisfactorily used on frozen and fixed material (Fig. 3E). Next, we moved to proteomics identification. This batch had higher numbers of protein identifications overall than the others (Supplementary Fig. S3A versus Supplementary Fig. S2A), however only the fixed sample resulted in a higher number of enriched proteins. The fresh nuclei had 79 enriched proteins, the frozen 122, and the fixed 687, of which 32 were enriched in all conditions, and an additional 87 shared in 2 out of the 3 (Fig. 3F). SMARCA4 was significantly enriched in all datasets, as well as other SWI/SNF components such as ARID1A/1B and SMARCE1, indicating that all tests were successful in identifying the expected interactors (Fig. 3F). We were curious whether the large difference obtained in fixed samples could be due to stabilization of protein complexes—indicating that fixation might be preferable for the proteomics portion of CUT&ID—or simply to an increased background. We used STRING [27] to search the list of fixed-only hits for high confidence (confidence-score 0.7) physical interactors of SMARCA4. We found nine known interactors; of these, only three were unique, while six of the nine had been found enriched in the first (unfixed) round of CUT&ID (from Fig. 2). We conclude that while fixation might slightly increase recovery, it is not strictly necessary for efficient interactome identification. We cannot rule out that fixation might be helpful for more rare or transient interactors, or when targeting particularly elusive proteins, and suggest *ad hoc* testing.

We tested CUT&ID for six additional proteins to cover the spectrum of possible chromatin-relevant targets: chromatin marks (H3K27ac), TFs (TCF7L2 and JUN), and several challenging non-DNA-binding co-factors (β-catenin, RAD21, and HDAC1). CUT&ID worked for all targets, yielding robust genomic and proteomic datasets that align well with existing data and known attributes for the targets (Fig. 4A–F). H3K27ac gave 17 445 genomic peaks and 301 enriched proximal proteins, where prominent were histone H3 (Log_2_FC 5.8, adj. *P* 2.5 × 10^−3^) and many chromatin regulators (Fig. 4A). TCF7L2 yielded in 24 273 genomic peaks and 36 high-confidence protein interactors, among which at the top were TCF7L2 (Log_2_FC 4.5, adj. *P* 4.5 × 10^−3^), β-catenin, and JUNB. Genomics and proteomics identifications were in line with current knowledge [28] and with each another—TCF7L2 and JUNB protein identification matched the enriched consensus motifs for TCF/LEF (*P* 1 × 10^−502^) and JUNB (*P* 1 × 10^−1836^) (Fig. 4B). β-Catenin was found to occupy 6282 genomic regions, of which 5970 (95%) overlapped with those bound by TCF7L2, as expected based on Wnt signaling mechanisms [28]. The top proteomic hit was β-catenin itself (Log_2_FC 6.1, adj. *P* 6.9 × 10^−5^) together with the members of the adherens junctions α-catenin and CDH1 (Fig. 4C)—indicating cytoplasm/membrane contamination during the nuclear extraction, which likely sequestered much of the antibody. The antibody was also more diluted than the others, and 0.4 μg instead of 2 μg were used. For JUN we obtained 4239 genomic peaks (top motif JUN/AP1 *P* 1 × 10^−204^), and 890 enriched proteins, with top hits of JUND (Log_2_FC 4.5, adj. *P* 2.6 × 10^−3^) and JUNB (Log_2_FC 3.8, adj. *P* 2.6 × 10^−3^), as well as reciprocal enrichment of TCF7L2 (Fig. 4D), cross-validating the TCF7L2 experiment. Previously, we and others had reported enriched motifs of JUN within TCF7L2 and β-catenin datasets; now, with the advantage of the proteomics readout of CUT&ID, we can confidently claim that they are truly co-binding—sitting on the same regulatory elements in single cells, something typically difficult to pinpoint in bulk datasets. RAD21 resulted in 6133 genomic peaks (top motif CTCF, *P* 1 × 10^−2274^) and closely matched the CTCF datasets (Supplementary Fig. S1). Note that we previously had to perform crosslinking to obtain RAD21 CUT&RUN data [29]: CUT&ID did not require crosslinking even to detect this elusive target, however fixation may be helpful to identify more peaks and transient DNA associations. We identified 51 high-confidence protein partners, including RAD21 (Log_2_FC 3.8, adj. *P* 1.8 × 10^−3^), and other known interactors like STAG1, STAG2, SMC1A, and SMC3 (Fig. 4E) [23]. Lastly, we obtained 11 466 HDAC1 peaks and 614 vicinal proteins, including HDAC1 (Log_2_FC 4.3, adj. *P* 5.8 × 10^−4^), and members of histone de-acetylation complexes such as SIN3A and DNTTIP1 [30] (Fig. 4F).

**Figure 4.**
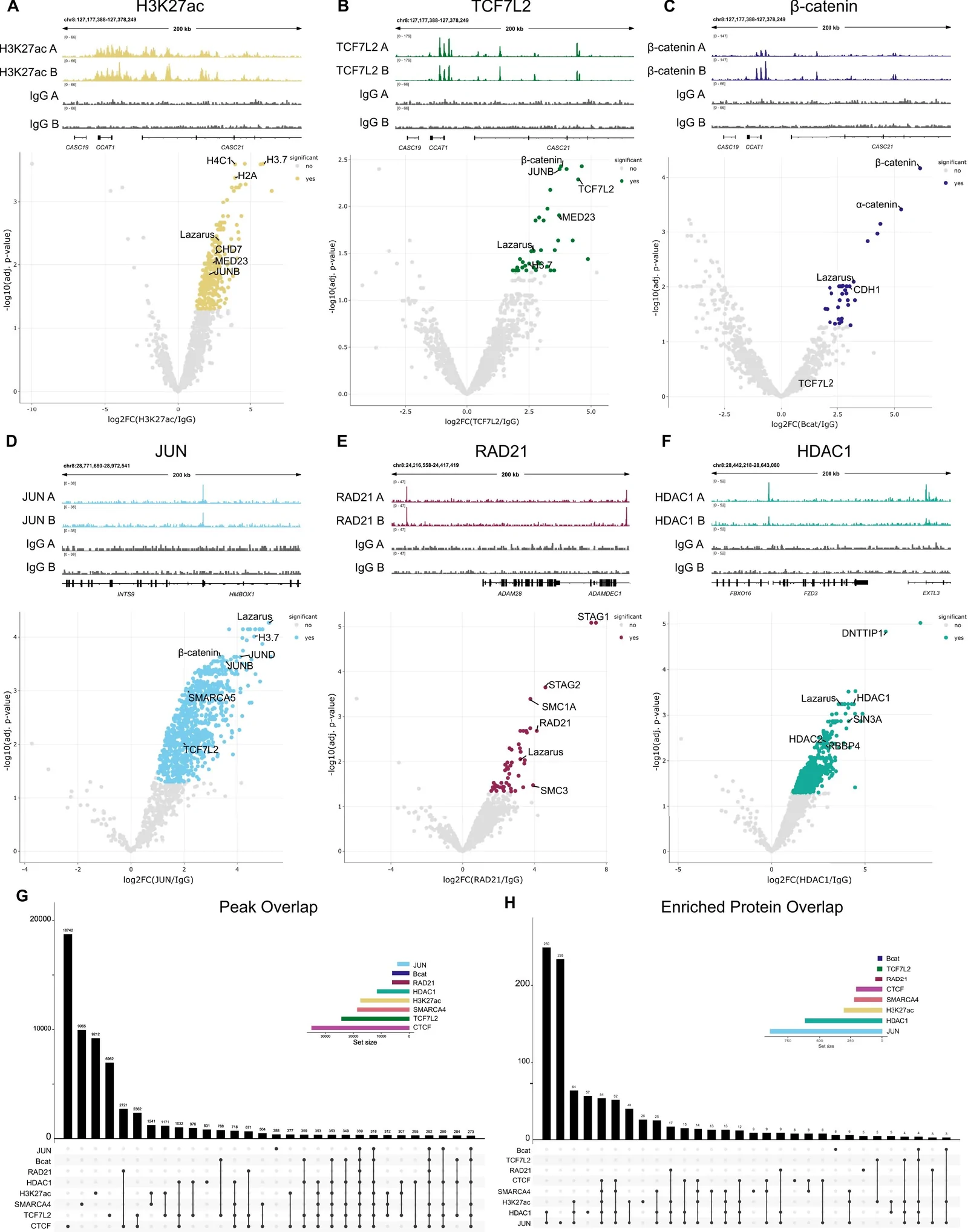
Applying CUT&ID to gene regulators. IGV Browser view (top) and volcano enrichment of proteins (bottom) using CUT&ID of (**A**) H3K27ac, (**B**) TCF7L2, (**C**) β-catenin, (**D**) JUN, (**E**) RAD21, and (**F**) HDAC1. Examples of known interactors are labeled. (**G**) Upset plot of peak overlap for all eight gene regulators profiled (including the CTCF and SMARCA4 data from Fig. 2). (**H**) Upset plot of enriched protein overlaps for all eight gene regulators. Peaks were called on merged replicates with MACS2 *q* < 0.05. Genomic datasets were normalized by reads per genome coverage and group scaled. Proteomic significance calculated using amica on default settings, with Log_2_FC > 1, adj. *P* < .05.

Next, we considered the overlap of the peaks and enriched proteins in all eight targets (including CTCF and SMARCA4 from Fig. 2) as a way to interrogate the precision of CUT&ID (Fig. 4G and H). The peaks followed expected patterns with many overlaps, as all our targets are expected to occupy some of the same regulatory elements according to their known functions. We saw especially high overlaps between known partners such as RAD21 and CTCF, or TCF7L2 and β-catenin, as described above. There were 339 regions called in all datasets, which might represent highly active enhancers. While overall protein identifications had greater than a 70% overlap between all datasets in each batch (Supplementary Figs S2G and S3G), the only protein enriched over the IgG in all targets was Lazarus itself, speaking in favor of the specificity of the technique and of the functionality of the IgG to control for abundant background proteins. While all the targets had overlapping binding, TurboID has a short range of 10 nm, and thus we expected more variation in the interactomes than we would see in the genomics. Furthermore, the number of enriched proteins did not seem to be reflective of the number of identified proteins: for example, H3K27ac and RAD21 both had ∼1000 identified proteins per replicate (Supplementary Fig. S2A), yet 301 were enriched in H3K27ac, and only 51 in RAD21. Likewise, the repeat SMARCA4 experiment (from Fig. 3) generally had more identifications, but not more enriched proteins, indicating that while batch effects exist—and thus controls should always be run in parallel with experimental samples—CUT&ID yields robust and reproducible top hits.

Taken together, these results show that CUT&ID is widely applicable to nuclear targets and is on par with individual state-of-the-art methods for genomics (ChIP-seq, CUT&RUN) and proteomics (PLAM-seq, ProtA-Turbo), yielding a dual omics readout in one shot with no sample splitting, no endogenous tagging nor overexpression, and offering the possibility to work on fresh, frozen, and fixated samples. These make CUT&ID uniquely versatile and applicable, opening the door for use in diverse experimental contexts, such as patient biopsies, which have been historically challenging for these types of assays.

## Discussion

CUT&ID emerges as a versatile and simple method to simultaneously map DNA binding and proximal protein interactions from the same sample and procedure. CUT&ID is robust, reproducible, and broadly applicable across histone modifications, TFs, and difficult-to-detect non-DNA-binding cofactors. We observed strong recovery of expected genomic features, including high signal-to-noise ratio, appropriate motif enrichment, and overlap with published reference datasets. Additionally, direct comparisons under the same experimental conditions confirmed that CUT&ID performs indistinguishably from CUT&RUN. In parallel, protein proximity labeling reliably identifies known interactors, with the bait protein invariably enriched at the top of the list, a necessary internal control of selective identification. The dual readout of CUT&ID also provides a unique opportunity to evaluate antibody specificity: proteins identified by mass spectrometry provide an orthogonal quality check for the genomic dataset, revealing whether off-target antibody binding might contribute to unexpected peaks or interactions. Native conditions, such as those used in CUT&ID, may reveal different off-targets compared to the denaturing buffers required in western blot. This built-in validation increases confidence in both the antibody and the specificity of the resulting datasets, an important consideration for the community studying gene regulation.

CUT&ID has several advantages over existing methods. It does not require cloning and overexpression nor endogenous knockins (as in BioID/TurboID [7] or PLAM-seq [9]), making it readily employable for any endogenous targets across models, *ex vivo* tissues, and even patients’ samples. It does not require crosslinking of cells or nuclei (like in ChIP-seq [2], PLAM-seq [9], and RIME [6]), reducing the introduction of undesired artifacts and minimizing the variability across experiments caused by differences in fixation. But CUT&ID is compatible with fixation, important for more fragile tissues or dynamic targets, and with flesh-freezing, opening up the possibility of using this method on biobanks of tissue that have been carefully stored in the past. It uses input material comparable to CUT&RUN [3], and considerably less than ChIP-seq [2]. Lastly, unlike ProtA-Turbo [8] CUT&ID’s streamlined workflow allows for dual output without sample splitting, and works via CUT&RUN, while the latter only tested compatibility with ChIP-seq.

CUT&ID is designed with scalability in mind, and a portion of its innovation lies in its streamlined protocol. The workflow uses low volumes, multichannel pipetting, is compatible with parallel processing of dozens of samples, and is easily adaptable to 96-well plates or automation. Moreover, its chemistry and conditions (e.g. temperatures and incubation times) have been meticulously tested to work for both genomics and proteomics identification. This is important, as several of its steps and procedural principles, we believe and hope, may now be beneficially adapted to improve other comparable methods in both fields.

## Limitations

While we strived to test CUT&ID across the board of different targets with varying inherent difficulty (from chromatin marks to non-DNA-binding co-factors), the method may still benefit from optimization for different tissues or targets. For the samples presented here, we used 1 million cells per replicate, a relatively low default amount that allowed us to yield robust genomic and proteomic datasets across all targets tested. We realize that this requirement may still prevent CUT&ID from being used on very rare cell types. We believe that optimal input may vary depending on various technical (e.g. antibody affinity) or biological (target abundance and dynamicity) variables. When targeting high-abundance proteins, or when using efficient antibodies, CUT&ID might be amenable to scaling down considerably further, as much as sensitivity in peptide-detection technologies allows. On the other hand, increasing input or number of replicates could enable the detection of more rare or transient interactions, or small, quantitative differences between experimental conditions. Other reaction conditions, such as biotinylation time or cleavage time and temperature, may also benefit from individual optimization. We recommend target-specific optimization based on these parameters and the biological question asked.

The use of an isotype IgG control antibody may sometimes appear as problematic given the unpredictable nature of what the uncharacterized mixture of antibodies it contains may target. In principle, IgGs should generate a background based on non-specific antibody binding, as well as controlling for off-target biotinylation that might occur during the protocol—for example during the Lazarus’ binding step or the washes—though this should be minimal due to the buffer conditions. In our hands, accordingly, the IgG sample was an overall reliable control. Here, we found that the DNA cleavage appeared mostly random, homogenously distributed along the genome, and the biotinylation hit highly abundant nuclear proteins and the usual contaminants, indicating no other bias. A certain level of background is even helpful, as imputation may inflate fold changes. As antibodies can have off-target binding, different levels of affinity and target different epitopes, it might be worthwhile, in addition to IgG, to include multiple antibodies for the same target. However, as with all other antibody-based assays, the ideal control is using the same antibody in a knock-out cellular context that *de facto* lacks the primary target.

We noticed that more abundant targets (for example, H3K27ac) naturally resulted in identification of more proximal proteins. This may very well include proteins that are common in the nuclei but are not functionally relevant interactors. It is also likely that proteins that have interactions both in the nucleoplasm and on the chromatin may result in more interactors, which are not necessarily chromatin associated, something to keep in mind when interpreting results. Non-DNA-binding proteins, like HDAC1, RAD21, and β-catenin, showed less consistency between the replicates on the genomic level, in line with our previous findings that more dynamic targets may require more replicates to detect the full spectrum of binding events [31]. As with other methods, results from CUT&ID will benefit from orthogonal validation and functional testing to determine biological relevance of the detected regulatory region binding and protein interactions.

## Supplementary Material

gkag943_Supplemental_Files

## Data Availability

Raw sequencing data and bigwigs for visualization have been deposited at ArrayExpress (E-MTAB-16382). Raw proteomics data and .sne files from Spectronaut analysis have been deposited at ProteomeXchange (PXD071610).

## Notes

Present address: Department of Biochemistry and Molecular Biology, Functional Genomics & Metabolism Unit, Southern Denmark University, DK-5230 Odense, Denmark
