# Supplementary material for: CUT&ID for simultaneous profiling of protein–DNA and protein–protein interactions
Source: Nucleic Acids Res. 2026 Sep 30;54(18):gkag943. doi: 10.1093/nar/gkag943 (PMC13624661; doi:10.1093/nar/gkag943)
Supplement: Supplementary file 1 [file gkag943_supplemental_files.zip › SuppFiguresLegends.pdf]

Supplementary Figures and Legends

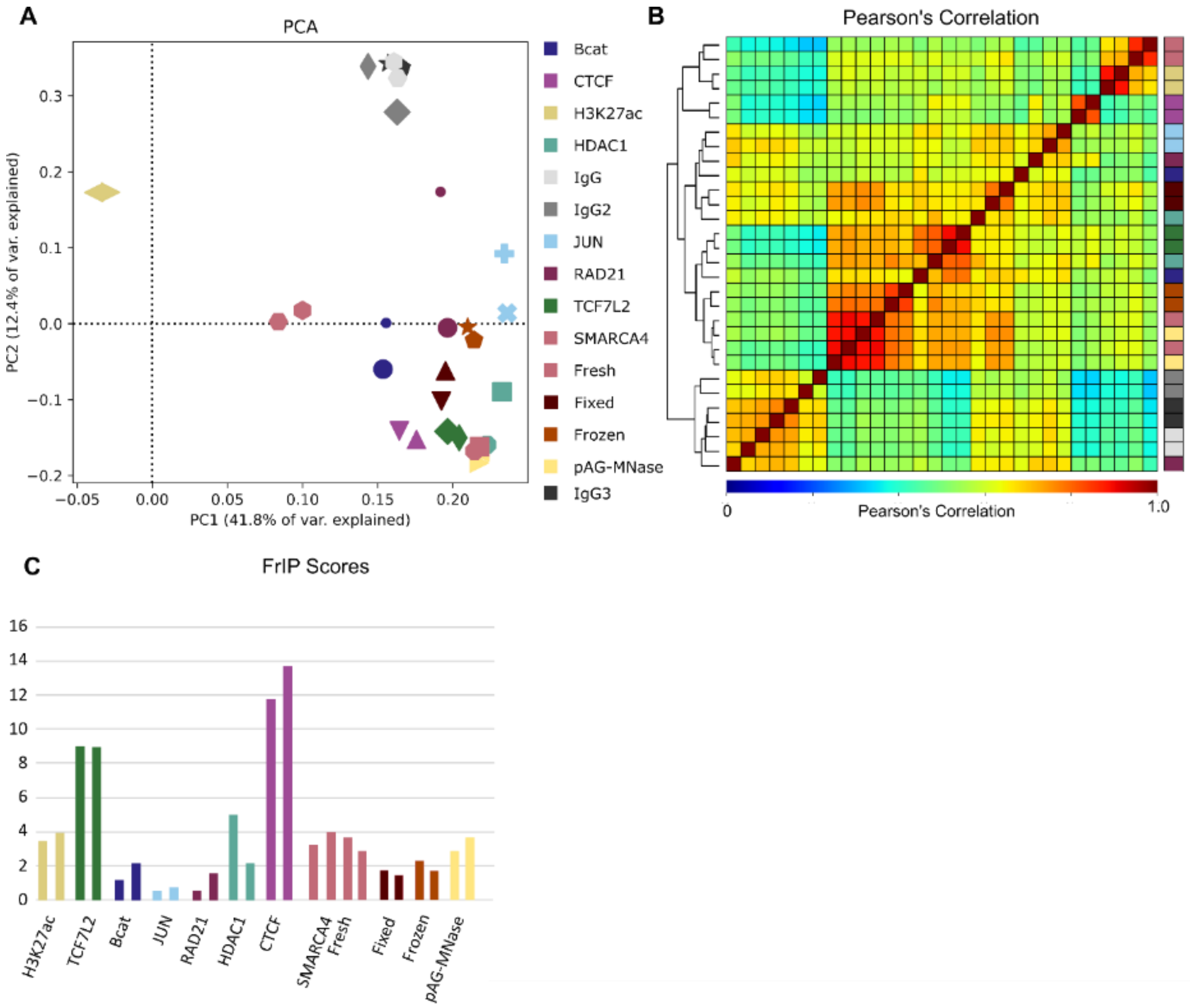

Figure S1. Quality control of genomic datasets. A. PCA of all samples done after genomic binning. IgG samples cluster together away from others **B.** Pearson's correlation of all samples after genomic binning. **C.** FrIP scores of all replicates, based on peaks called on merged replicates (MACS2 q < 0.05).

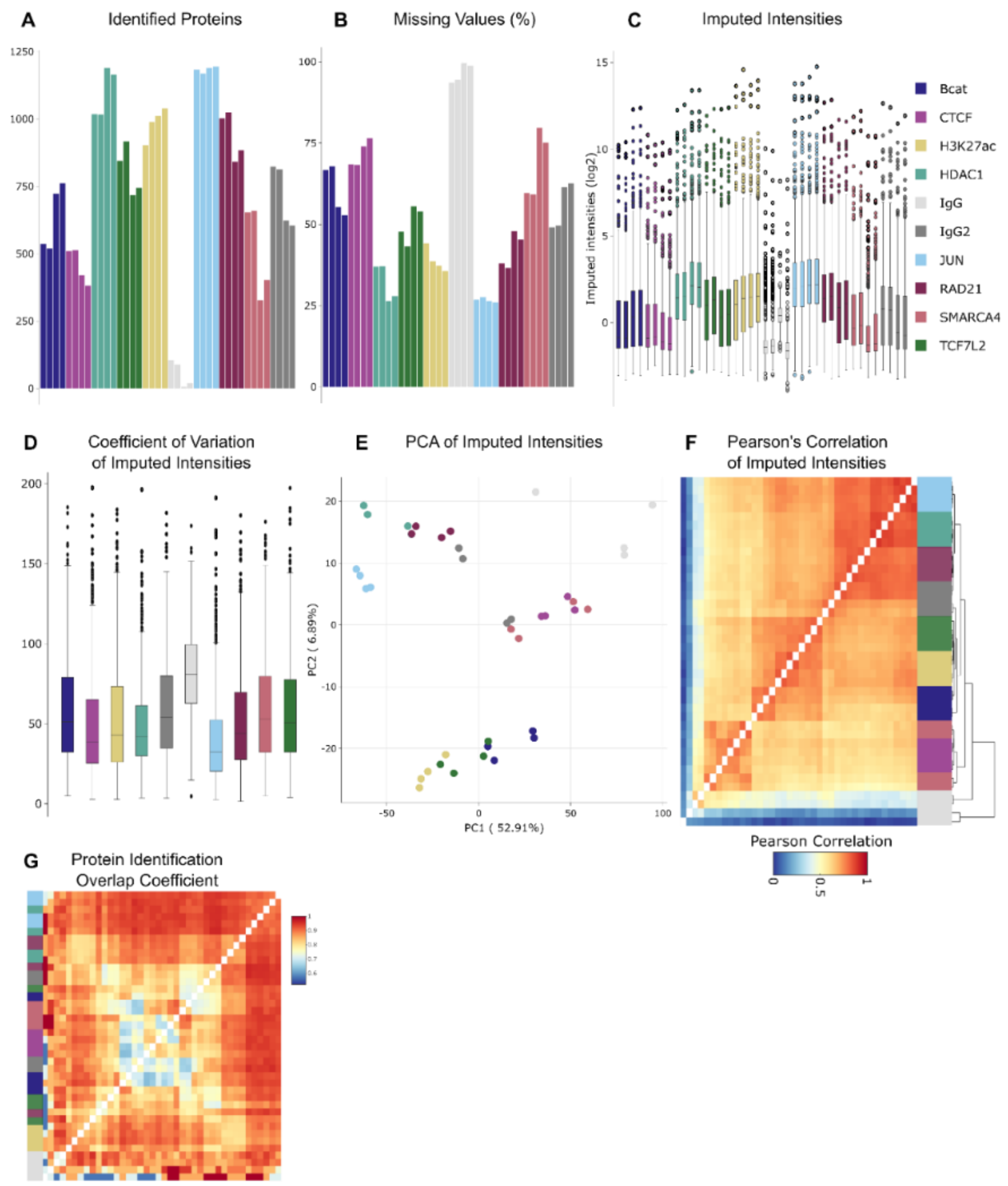

Figure. S2. Quality control of proteomic datasets for the eight gene regulators. A. Number of identified proteins in all replicates. CTCF and SMARCA4 were performed in parallel with IgG, and the rest with IgG2. Generally, the first experiment identified fewer proteins. **B.** Percentage of missing values in all datasets. **C.** Boxplot of imputed intensities for all samples. Imputation was performed with the amica software on default settings, after initial processing with Spectronaut. **D.** Coefficient of variation of imputed intensities for all targets. **E.** PCA of imputed intensities for all samples. **F.** Pearson's correlation of imputed intensities of all samples. **G.** Overlap coefficient of identified proteins of all samples. Samples in all plots are colored by target, legend in top right corner.

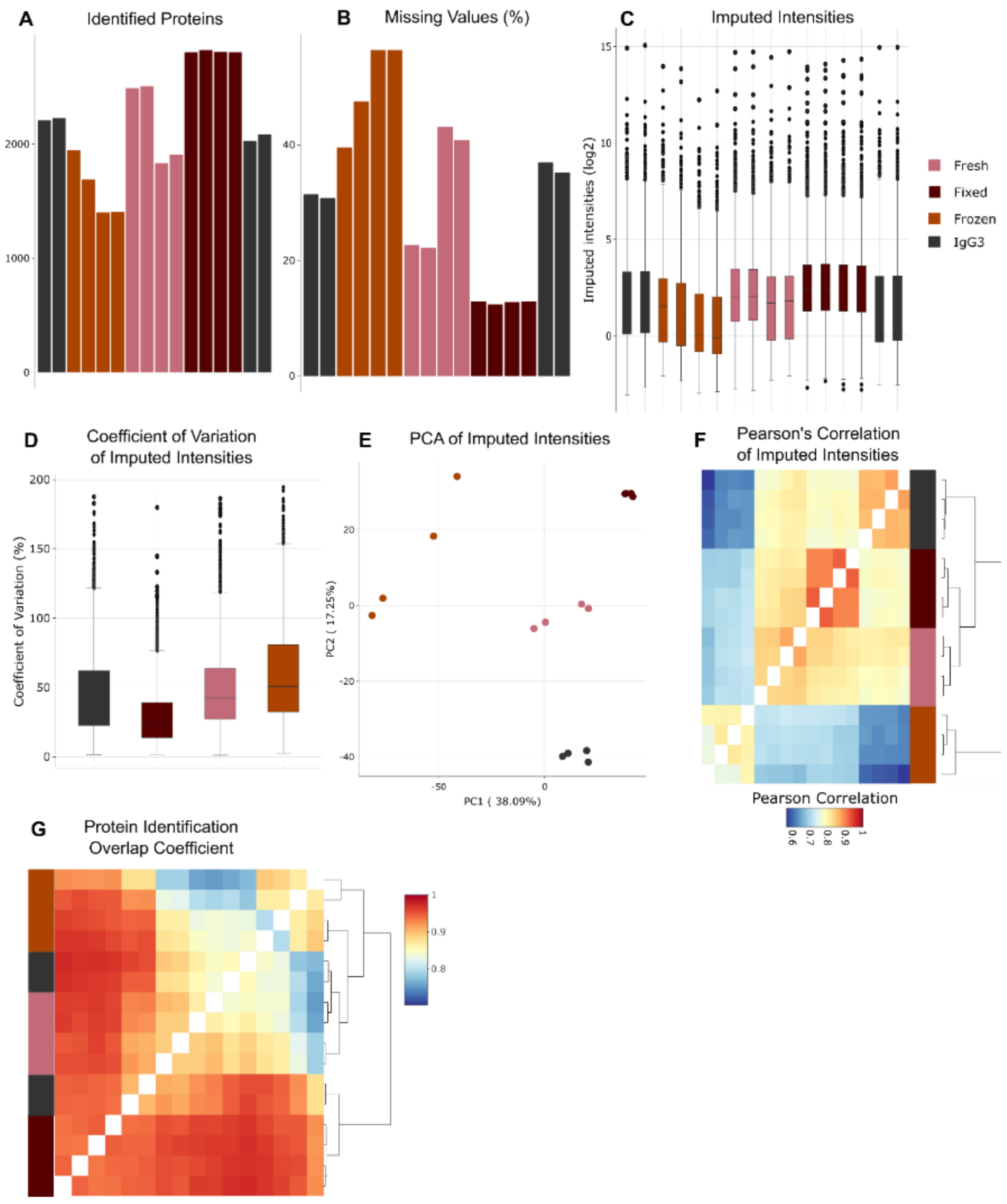

Figure S3. Quality control of proteomic datasets for fresh, fixed and frozen experiment. A. Number of identified proteins in all replicates. IgG was performed on fresh nuclei. **B.** Percentage of missing values in all datasets. **C.** Boxplot of imputed intensities for all samples. **D.** Coefficient of variation of imputed intensities for all targets. **E.** PCA of imputed intensities for all samples. **F.** Pearson's correlation of imputed intensities of all samples. **G.** Overlap coefficient of identified proteins of all samples. Samples in all plots are colored by condition, legend in top right corner.

Supp. File 1 contains a detailed, step-by step protocol for performing CUT&ID with troubleshooting tips.

Supp. Table 1 contains peak lists for all merged datasets.

Supp. Table 2 contains lists of enriched proteins for all datasets.

Supp. Table 3 contains values exported from Spectronaut analysis, including number of precursors, run evidence counts, and protein group amounts for all targets.

Supp. Table 4 contains amica output data and sample information, which can be reuploaded to the amica online portal for data exploration and visualization.
